# Mapping the research landscape of artificial intelligence in heart failure: a bibliometric analysis

**DOI:** 10.1097/MS9.0000000000005235

**Published:** 2026-07-03

**Authors:** Pegah Rashidian, Saisree Reddy Adla Jala, Kavya Priya Somu, Abinash Mahapatro, Nikitha Chellapuram, Seyyed Mohammad Hashemi, Abdulhadi Jameel Alotaibi, Omar Hasan Hasan, Amir Nasrollahizadeh, Negin Letafatkar, Maryam Jafari, Nimra Shafi, Ehsan Amini-Salehi

**Affiliations:** a Vali-e-Asr Reproductive Health Research Center, Family Research Institute, Tehran University of Medical Sciences, Tehran, Iran; b Mission Hospital, Asheville, NC, USA; c Centinela Hospital Medical Center, Inglewood, CA, USA; d SIU School of Medicine, Springfield, IL, USA; e Cooper University Hospital, Camden, NJ, USA; f Cardiovascular Research Center, Hormozgan University of Medical Sciences, Bandar Abbas, Iran; g Department of Medicine, Vision Colleges, Riyadh, Saudi Arabia; h Tehran Heart Center, Cardiovascular Diseases Research Institute, Tehran University of Medical Sciences, Tehran, Iran; i Gastrointestinal and Liver Diseases Research Center, Guilan University of Medical Sciences, Rasht, Iran; j Department of Medicine, Arnot Ogden Medical Center, Elmira, NY, USA; k School of Medicine, Guilan University of Medical Sciences, Rasht, Iran

**Keywords:** artificial intelligence, cardiovascular systems, heart failure, machine learning, risk stratification

## Abstract

Heart failure (HF) is a complex syndrome with high morbidity and mortality. Despite advancements in treatment, its management remains a challenge. The objective of this study was to map the scientific landscape of artificial intelligence (AI) applications in HF management through a bibliometric analysis. Data were retrieved from the Web of Science Core Collection. Keywords related to AI and HF were used to identify relevant research articles. Various bibliometric tools, such as Biblioshiny, VOS viewer, and CiteSpace, were used for quantitative trends, collaboration networks, and thematic areas, which were assessed. A total of 1332 studies were included in the final analysis. Publication trends show a sharp increase in AI research related to HF from 2016 onward, with 317 studies published in 2025. The most frequent keywords in the field were *heart failure* (*n* = 599), *machine learning* (*n* = 516), and *AI* (*n* = 225). Other significant keywords included *mortality* (*n* = 201), *diagnosis* (*n* = 168), and *risk* (*n* = 162). Cluster analysis identified major research themes, including Cardiac & Cardiovascular Systems, Computer Science – Interdisciplinary Applications, Computer Science – Artificial Intelligence, Health Care Sciences & Services, Radiology, Nuclear Medicine & Medical Imaging, Cell Biology, Medical Informatics, Endocrinology & Metabolism, and Neurosciences. AI has rapidly become a central tool in HF management, with significant contributions from leading countries and institutions. However, further global collaboration and standardized reporting frameworks are needed to ensure the equitable translation of these technologies into clinical practice.

## Introduction

Heart failure (HF) is a complex clinical syndrome characterized by the heart’s inability to pump blood sufficiently to meet the body’s metabolic needs^[^1–3^]^. It affects an estimated 64 million people globally and remains one of the leading causes of hospitalization, morbidity, and mortality^[^4–6^]^. Despite advances in pharmacological and device-based therapies, effective management of HF continues to present major challenges^[^7–10^]^.

HIGHLIGHTS

- This bibliometric analysis highlights the rapid growth and emerging trends in AI research for heart failure, with a notable surge in publications since 2016.
- The study identifies key research themes, including predictive modeling, mortality prediction, and risk stratification in heart failure.
- AI’s increasing role in the early diagnosis and personalized care of heart failure is emphasized, showcasing its potential to improve clinical outcomes.

Artificial intelligence (AI) has recently emerged as a transformative tool in cardiology^[^11,12^]^. Applications of AI in HF management include predictive modeling for disease course, hospitalization, and mortality; advanced image interpretation for cardiac function and structural abnormalities; natural language processing for extracting insights from clinical records; and integration with wearable devices for remote monitoring of patients^[^13–21^]^. These technologies support earlier diagnosis, risk stratification, and proactive intervention, all of which hold the potential to improve patient outcomes^[^22–27^]^.

Bibliometric studies are increasingly recognized as powerful tools for assessing scientific progress across different fields of medicine. By applying quantitative techniques to large volumes of bibliographic data, they allow researchers to evaluate publication outputs, citation impact, and collaboration networks in a systematic way. This type of analysis provides a clearer understanding of the intellectual structure of a field, highlights the most influential contributors, and reveals the geographical and institutional distribution of research efforts^[^28–30^]^.

In the context of AI in HF, bibliometric analysis is particularly valuable given the rapid growth of publications and the highly interdisciplinary nature of the field^[^31–33^]^. This study aims to map the scientific landscape of AI in HF management using established bibliometric tools.

## Methods

Data for this bibliometric analysis were retrieved from the Web of Science Core Collection, which provides extensive coverage of peer-reviewed scientific literature and is widely used in bibliometric studies^[^34–36^]^. The search covered the period from the database’s inception until 11 September 2025, ensuring that both early research and the most recent publications were included. The strategy combined keywords related to AI (“artificial intelligence,” “machine learning,” “deep learning”) and HF (“heart failure,” “cardiac failure”), applied to titles, abstracts, and author keywords. Only peer-reviewed original articles and review papers published in English were included, while conference proceedings, letters, and editorials were excluded to maintain consistency and quality of the dataset. The exact search strategy used in this study is presented in Supplemental Digital Content Table S1, available at: http://links.lww.com/MS9/B270. The bibliographic records, including full metadata and cited references, were exported in plain text format. Data cleaning involved removing duplicates and verifying the completeness of author, title, and keyword information.

Quantitative trend analysis, including annual publication outputs and cumulative growth, was performed using Microsoft Excel. Further bibliometric performance analysis of journals, authors, institutions, and countries was conducted with Biblioshiny (version 5.1.0), the web-based interface of the R package Bibliometrix.

Structural and thematic mapping was carried out using VOSviewer (version 1.6.20), which generated co-authorship, co-citation, and keyword co-occurrence networks. Density and overlay visualizations were applied to highlight emerging research areas. In addition, CiteSpace (version 6.4.R2) was used to detect citation bursts, identify research frontiers, and visualize temporal evolution of the field.

The combined use of these tools enabled a comprehensive evaluation of the scientific output, collaborative structures, and knowledge evolution in the field of AI in HF management.

## Results

**Figure 1.**
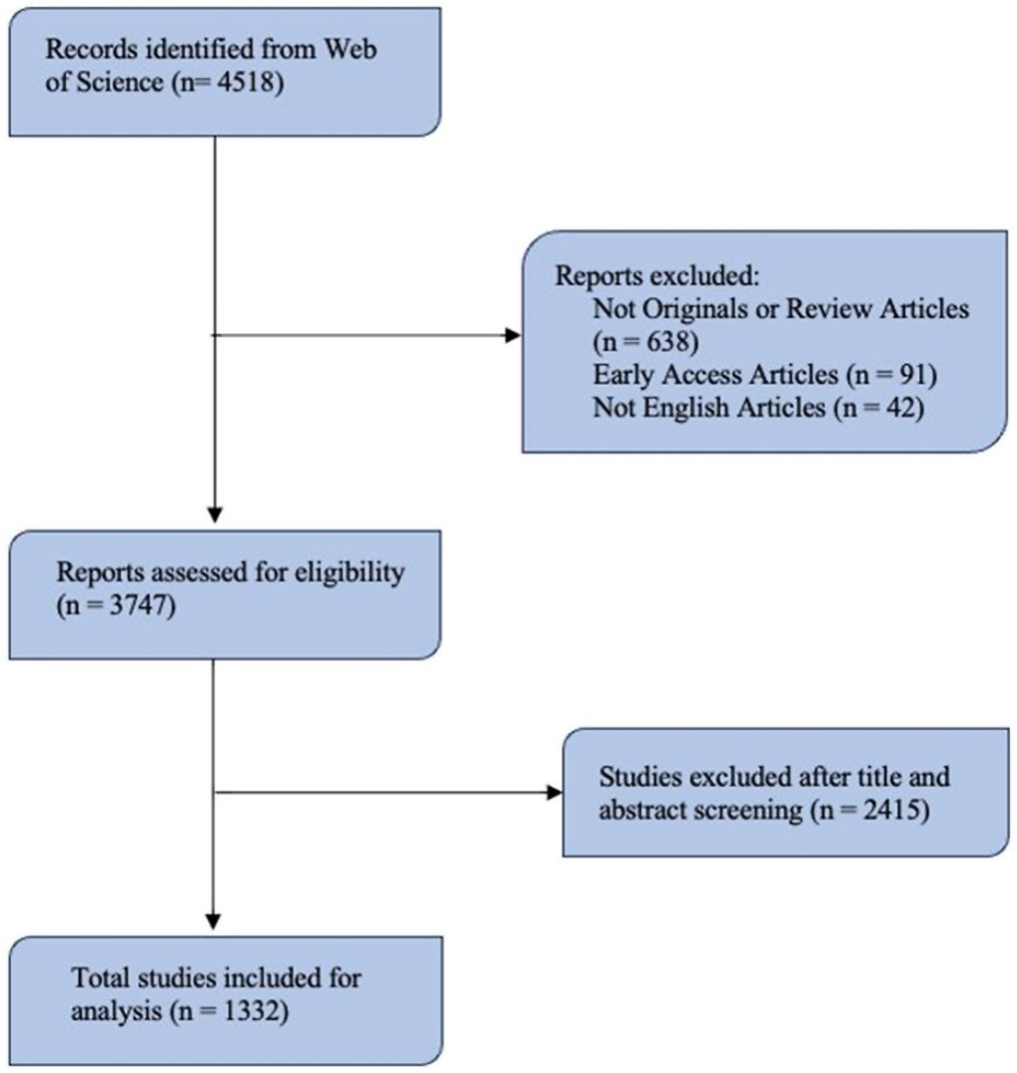
Study selection process.

A total of 4518 records were initially identified from the Web of Science database. After the removal of reports that did not meet the inclusion criteria, such as articles that were not original research or review articles (638 reports), early access articles (91 reports), and articles not in English (42 reports), 3747 reports were assessed for eligibility. Following the title and abstract screening, a total of 2415 studies were excluded, leaving 1332 studies that were included in the final analysis (Fig. 1).

## Publication trend

**Figure 2.**
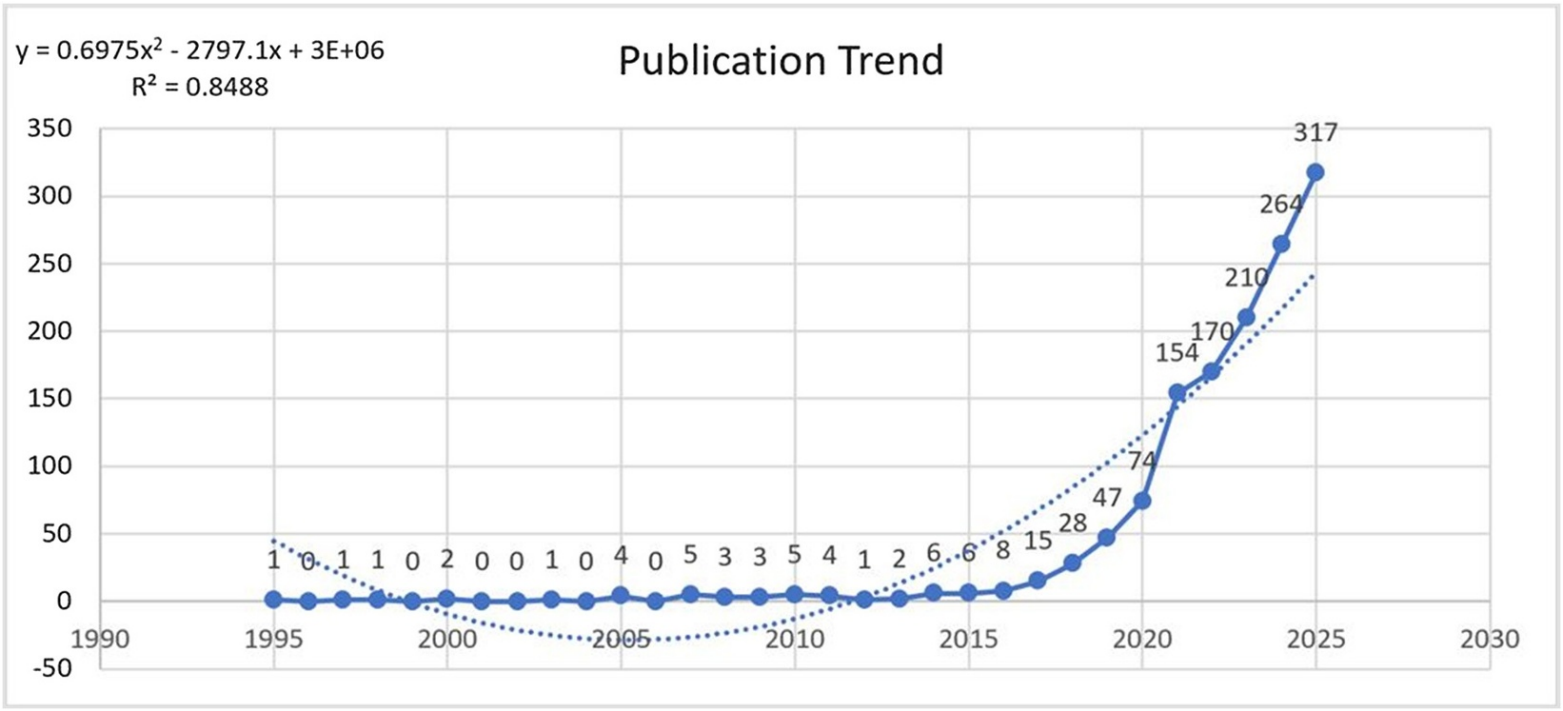
Annual trend in publication regarding AI in HF.

The development of research on AI in HF can be observed through the pattern of publications over time. During the early years, from 1995 through the early 2000s, research activity was limited, with only one or two studies published each year, and some years had no publications. From 2005 to 2015, there was a gradual increase, but the overall production remained relatively modest. A major shift occurred after 2016, when the number of studies began to rise rapidly. In 2016, 8 studies were published, and by 2018, this number had grown to 28. The upward momentum continued, with an even sharper increase over the following years, culminating in 154 studies in 2021, 264 in 2024, and 317 in 2025 (Fig. 2).

**Figure 3.**
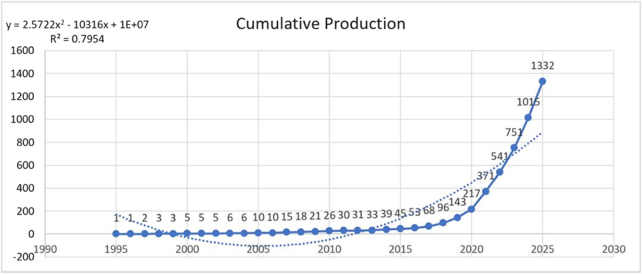
Cumulative publications regarding AI in HF.

The cumulative production of research in this area underscores this expansion even more clearly. From a single study in 1995, the total number of publications remained below 50 until 2015. After 2016, the cumulative count began to climb steeply, reaching 96 by 2018, 217 by 2020, and 751 by 2023. By 2025, the cumulative total had reached 1332 studies (Fig. 3).

## Leading countries

The examination of global contributions revealed that 88 countries had collaborated in this research domain (Supplemental Digital Content Figure S1, available at: http://links.lww.com/MS9/B270). The analysis of the top ten countries by publication output revealed that the United States contributed the highest number of papers (*n* = 427), followed by China (*n* = 324) and England (*n* = 102). Japan (*n* = 82), Italy (*n* = 76), Germany (*n* = 65), the Netherlands (*n* = 63), Canada (*n* = 59), India (*n* = 57), and South Korea (*n* = 51) also made notable contributions to the field (Table 1).

**Table 1:** Top 10 countries by number of publications.

Rank	Country	Number of publications
1	USA	427
2	People’s Republic of China	324
3	England	102
4	Japan	82
5	Italy	76
6	Germany	65
7	Netherlands	63
8	Canada	59
9	India	57
10	South Korea	51

The top 10 countries in terms of centrality are presented in Table 2. The United States had the highest betweenness centrality (0.11), followed by Italy (0.10), India (0.09), and Saudi Arabia (0.09). France (0.08) and Singapore (0.06) also exhibited strong bridging roles in the network. Germany (0.05), Australia (0.05), and Slovenia (0.05) contributed significantly to the connectivity of the field, while Sweden (0.04) completed the list of the top ten countries by centrality. Supplemental Digital Content Figure S2, available at: http://links.lww.com/MS9/B270, illustrates the international collaboration network among countries, highlighting central nodes such as the USA.

**Table 2:** Top 10 countries by centrality.

Rank	Country	Centrality
1	USA	0.11
2	Italy	0.10
3	India	0.09
4	Saudi Arabia	0.09
5	France	0.08
6	Singapore	0.06
7	Germany	0.05
8	Australia	0.05
9	Slovenia	0.05
10	Sweden	0.04

## Journals and co-cited journals

The bibliometric analysis identified a wide range of journals publishing on AI in HF (Supplemental Digital Content Figure S3, available at: http://links.lww.com/MS9/B270). *Frontiers in Cardiovascular Medicine* led with (*n* = 59) publications, followed by *ESC Heart Failure* (*n* = 50) and *Scientific Reports* (*n* = 36). Other highly productive sources included *European Heart Journal Digital Health* (*n* = 29), *PLOS One* (*n* = 22), *Diagnostics* (*n* = 19), *European Journal of Heart Failure* (*n* = 18), and *IEEE Access* (*n* = 16). The *International Journal of Cardiology* (*n* = 16) and the *Journal of the American Heart Association* (*n* = 15) also made notable contributions to the literature.

The temporal analysis of publication trends across leading journals revealed a clear and rapid growth pattern (Supplemental Digital Content Figure S4, available at: http://links.lww.com/MS9/B270). From 1995 through 2015, the cumulative number of articles remained very low, with *PLOS One* being the only journal with early contributions. A gradual increase began after 2018, but the most dramatic rise occurred between 2020 and 2025. *Frontiers in Cardiovascular Medicine* showed the steepest trajectory, growing to 59 publications by 2025, making it the most productive journal in this field. *ESC Heart Failure* followed a similar upward trend, reaching 50 publications in 2025 (Supplemental Digital Content Figure S4, available at: http://links.lww.com/MS9/B270).

## Authors and co-cited authors

The analysis of contributions in the field of AI in HF identified several leading authors who have significantly shaped the research landscape. Sanjiv J. Shah emerged as the most prolific author, with (*n* = 15) publications, followed by Francisco Lopez-Jimenez (*n* = 14) and Paul A. Friedman (*n* = 13). Peter A. Noseworthy contributed to 11 publications, while Chin Lin, Chin-Sheng Lin, Javed Butler, and Gregg C. Fonarow each authored 10 publications.

In terms of total citations, Ambarish Pandey was the most-cited author with 674 citations, followed by Matthew W. Segar (*n* = 633) and Harlan M. Krumholz (*n* = 584). Gregg C. Fonarow (*n* = 556) and Francisco Lopez-Jimenez (*n* = 529) also ranked among the top five most-cited authors, highlighting their strong influence in this domain.

The co-citation analysis revealed that Shah SJ was the most frequently co-cited author (*n* = 99), followed by Ponikowski P (*n* = 90) and Chen (*n* = 64). CW Yancy (*n* = 57) and Leo Breiman (*n* = 56) also appeared as major knowledge sources, reflecting their foundational contributions to the field (Table 3).

**Table 3:** Top 10 authors by publications, total citations, and co-citations, reflecting their collective impact on the development of research in AI for heart failure.

Number	Author with high number of publications	Number of publications	Author with high number of citations	Number of citations	The most co-cited authors	Number of co-citations
1	Shah, Sanjiv J.	15	Pandey, Ambarish	674	Shah, Sj	99
2	Lopez-Jimenez, Francisco	14	Segar, Matthew W.	633	Ponikowski, P.	90
3	Friedman, Paul A.	13	Krumholz, Harlan M.	584	Chen	64
4	Noseworthy, Peter A.	11	Fonarow, Gregg C.	556	Yancy, Cw	57
5	Lin, Chin	10	Lopez-Jimenez, Francisco	529	Breiman, L.	56
6	Lin, Chin-Sheng	10	Friedman, Paul A.	462	Acharya, Ur	54
7	Butler, Javed	10	Noseworthy, Peter A.	453	Attia, Zi	48
8	Fonarow, Gregg C.	10	Shah, Sanjiv J.	434	Isler, Y.	40
9	Li, Yan	9	Vaduganathan, Muthiah	410	Kwon, Jm	38
10	Greenberg, Barry	9	Acharya, U. Rajendra	381	Ahmad, T.	37

## Top-cited papers

Supplemental Digital Content Table S2, available at: http://links.lww.com/MS9/B270, lists the 10 most-cited papers in the field of AI in HF, underscoring their substantial influence on the research landscape. The most-cited paper was authored by Choi E. and published in the *Journal of the American Medical Informatics Association* in 2017, receiving 528 citations. The second most-cited paper was by Wu J.L. in *Medical Care* (2010), with 264 citations, followed by Güler I.’s 2005 article in *Pattern Recognition* with 257 citations.

Mortazavi B.J.’s 2016 publication in *Circulation: Cardiovascular Quality and Outcomes* ranked fourth with 252 citations, while Frizzell J.D.’s 2017 paper in *JAMA Cardiology* closely followed with 251 citations. Chicco D.’s 2020 study in *BMC Medical Informatics and Decision Making* received 250 citations, highlighting its relevance. Other highly cited works included Yao X.X. (2021, *Nature Medicine, n* = 238), Paulus W.J. (2021, *Circulation Research, n* = 229), Masetic Z. (2016, *Computer Methods and Programs in Biomedicine, n* = 215), and Segar M.W. (2020, *European Journal of Heart Failure, n* = 210). Supplemental Digital Content Figure S5, available at: http://links.lww.com/MS9/B270, shows the citation burst of the articles.

## Keyword trends, hotspots, and cluster analysis

The keyword analysis revealed several core research themes in the field of AI in HF. The most frequently occurring keyword was HF (*n* = 599), followed by machine learning (*n* = 516) and AI (*n* = 225). Other high-frequency keywords included mortality (*n* = 201), diagnosis (*n* = 168), and risk (*n* = 162) (Supplemental Digital Content Figure S6, available at: http://links.lww.com/MS9/B270).

The analysis of central keywords, which highlights the most influential terms within the research network, identified the following top ten: “heart failure” (0.22), “classification” (0.19), “risk” (0.15), “association” (0.10), “death” (0.10), “machine learning” (0.09), “management” (0.08), “prediction” (0.08), “mortality” (0.07), and “care” (0.07) (Supplemental Digital Content Figure S7, available at: http://links.lww.com/MS9/B270).

The cluster analysis identified nine major research clusters, each representing a distinct thematic area within the field. These clusters included Cardiac & Cardiovascular Systems (#0), Computer Science – Interdisciplinary Applications (#1), Computer Science – Artificial Intelligence (#2), Health Care Sciences & Services (#3), Radiology, Nuclear Medicine & Medical Imaging (#4), Cell Biology (#5), Medical Informatics (#6), Endocrinology & Metabolism (#7), and Neurosciences (#8) (Supplemental Digital Content Figure S8, available at: http://links.lww.com/MS9/B270). Together, these clusters demonstrate the multidisciplinary nature of AI in HF research, integrating clinical, computational, and imaging domains.

## Discussion

HF remains a major global health challenge, contributing substantially to morbidity, mortality, and healthcare expenditures despite advances in pharmacological and device-based therapies. Conventional strategies for diagnosis, risk stratification, and management often face limitations related to delayed recognition, heterogeneity of clinical presentations, and restricted capacity for individualized care. Against this backdrop, AI has emerged as a transformative technology with the potential to bridge these gaps. By leveraging large-scale clinical, imaging, and biomarker data, AI provides novel opportunities for earlier detection, improved prognostic modeling, and personalized therapeutic strategies. The findings of this bibliometric analysis demonstrate that research activity on AI in HF has expanded dramatically in recent years, underscoring its growing recognition as a pivotal tool to address longstanding challenges and reshape the future of cardiovascular care.

The publication trajectory of AI in HF research reveals a clear transition from a nascent to a rapidly expanding field. The limited output prior to 2015 reflects the early exploratory phase, when computational capabilities and data availability constrained large-scale applications. The sharp increase after 2016 coincides with breakthroughs in machine learning and deep learning, coupled with the integration of electronic health records and advanced imaging datasets, which collectively enabled more clinically relevant investigations. The exponential rise in studies after 2020, culminating in over 300 publications in 2025, underscores the growing recognition of AI as a critical tool in addressing the global burden of HF. This surge highlights both the maturation of AI methodologies and the increasing prioritization of digital health solutions, positioning AI as a cornerstone in the evolution of cardiovascular care.

The global distribution of research on AI in HF demonstrates a marked imbalance, with the United States and China emerging as the leading contributors. The United States not only generated the largest number of publications but also achieved the highest centrality score, underscoring its pivotal role in shaping scientific agendas and fostering international collaboration. China’s rapid growth in research output reflects its expanding presence in digital health and cardiovascular innovation, while countries such as England, Japan, Italy, and Germany have also provided substantial contributions, reinforcing the prominence of established research systems in this domain. The bridging roles observed for nations including India, Saudi Arabia, and France highlight their importance in facilitating cross-border collaborations and indicate a gradual diversification of the research landscape. Nonetheless, the concentration of scholarly activity within a limited group of countries underscores ongoing disparities in research capacity, which may constrain the global applicability and integration of AI-driven approaches in HF.

The examination of the most frequently cited references highlights several landmark studies that have laid the foundation for advancing AI applications in HF, providing important insights into early diagnosis, prognostic modeling, and personalized management. The most-cited study in this field is by Edward Choi and colleagues, entitled “Using recurrent neural network models for early detection of HF onset” (2017). In this landmark work, the authors analyzed electronic health record (EHR) data from 3884 incident HF cases and 28 903 matched controls, applying advanced deep learning techniques – specifically recurrent neural networks with gated recurrent units (GRUs) – to capture temporal patterns in clinical events. Their AI-driven model significantly outperformed conventional approaches such as logistic regression, multilayer perceptrons, support vector machines, and k-nearest neighbors, achieving an AUC of 0.777 with a 12-month observation window and 0.883 with 18 months, compared with a maximum of 0.834 for baseline methods. The study concluded that harnessing AI to model temporal dynamics in longitudinal EHRs enables more accurate and earlier prediction of HF onset, underscoring the transformative role of AI in risk stratification and clinical decision support for cardiovascular care.

The second most-cited study in this field is by Wu *et al*, titled “Prediction Modeling Using EHR Data: Challenges, Strategies, and a Comparison of Machine Learning Approaches.” This work analyzed electronic health record data from the Geisinger Clinic, including 536 HF cases and nearly 4000 matched controls, to evaluate whether machine learning could predict HF more than 6 months before clinical diagnosis. The authors compared logistic regression, support vector machines (SVM), and Boosting, applying variable selection strategies and 10-fold cross-validation to assess robustness. Their results demonstrated that both logistic regression and Boosting achieved strong performance (AUC ≈ 0.76–0.77), while SVM underperformed, likely due to challenges with imbalanced and mixed data types. Importantly, the study highlighted that machine learning applied to longitudinal EHR data can anticipate HF onset months before clinical recognition, underscoring the potential of AI-based predictive models to support earlier intervention and improve outcomes in routine care.

The third most-cited study, by İnan Güler and Elif Derya Übeyli, titled “ECG Beat Classifier Designed by Combined Neural Network Model” (2005), focused on the development of an automated approach for classifying electrocardiogram (ECG) signals. Using data from the PhysioBank database, the authors analyzed four categories of beats – normal, congestive HF, ventricular tachyarrhythmia, and atrial fibrillation – by applying discrete wavelet transform (DWT) for feature extraction, followed by a two-level combined neural network architecture. The first level of neural networks classified beats using statistical features derived from wavelet coefficients, and the outputs were then integrated by a second-level network to enhance diagnostic accuracy. This ensemble framework achieved an overall accuracy of 96.94%, outperforming stand-alone neural network models. The study concluded that combining neural networks with time–frequency analysis can substantially improve the reliability of automated cardiac diagnostics, underscoring the potential of AI approaches to support clinical decision-making in HF and arrhythmia detection.

The fourth most-cited study, by Mortazavi *et al* (2016), titled “Analysis of Machine Learning Techniques for HF Readmissions,” evaluated data from 1653 patients in the Tele-HF trial to compare machine learning models with logistic regression for predicting readmissions. Logistic regression showed poor discrimination (C-statistics 0.53–0.57), while random forests and boosting provided modest improvements, particularly for 30-day outcomes. The study concluded that, although machine learning does not achieve perfect prediction, it offers meaningful gains over traditional methods and highlights the potential of AI to improve risk stratification in HF. Collectively, these highly cited studies have established the foundation for applying AI to HF by demonstrating its potential in early diagnosis, risk prediction, patient stratification, and readmission forecasting. Although results vary in performance, together they highlight both the promise and the limitations of current approaches, underscoring the importance of integrating richer datasets, advanced algorithms, and multidisciplinary collaboration to translate AI innovations into meaningful clinical impact.

In the context of HF, the frequent occurrence of the keywords mortality, diagnosis, and risk underscores the major clinical challenges where AI can deliver meaningful impact. AI-based models have shown the ability to enhance mortality prediction by identifying high-risk patients through complex clinical, imaging, and biomarker patterns, thereby enabling earlier and more targeted interventions. For example, Lee and colleagues demonstrated that deep learning models trained on ECG data could substantially improve 1-year mortality prediction in patients with reduced ejection fraction, reinforcing the potential of AI to reduce mortality risk and support personalized management strategies^[^37^]^. Similarly, Kwon and colleagues developed a deep-learning algorithm (DAHF) for acute HF that was trained on more than 12 000 cases and validated in nearly 5000 patients. Their model significantly outperformed conventional risk scores such as GWTG-HF and MAGGIC, accurately stratifying patients into high- and low-risk groups, further highlighting the value of AI in advancing prognostic assessment and clinical decision-making in HF^[^38^]^.

Similarly, AI enhances diagnostic accuracy, overcoming the limitations of conventional approaches by leveraging advanced analyses of ECG, echocardiography, cardiac MRI, and even wearable device data to support earlier and more reliable detection of HF. This was further demonstrated in a recent systematic review and meta-analysis by Ferreira and colleagues, which confirmed that AI-enabled ECG algorithms achieve high sensitivity and specificity for detecting reduced ejection fraction, underscoring their value as a noninvasive and accessible diagnostic tool^[^39^]^. Recent reviews have increasingly underscored the pivotal role of AI in enhancing the diagnostic accuracy of HF^[^40,41^]^.

Furthermore, AI-driven approaches to risk stratification offer more personalized prognostic assessments by integrating diverse patient variables, thereby informing individualized management plans and reducing adverse outcomes. In this context, Dhingra and colleagues demonstrated that a noise-adapted AI-ECG model trained on single-lead recordings could effectively predict left ventricular systolic dysfunction and subsequent HF risk in large multinational cohorts, including over 190 000 patients. A positive AI-ECG screen was associated with a 3- to 7-fold increased risk of developing HF, and incorporating this model alongside traditional risk scores (PCP-HF and PREVENT) significantly improved discrimination and reclassification^[^42^]^. Additionally, Butler and colleagues developed an ECG-based AI model for 10-year HF risk prediction that significantly outperformed traditional calculators (ARIC-HF and FHS-HF), with combined models achieving AUCs up to 0.84. Importantly, the algorithm demonstrated strong predictive accuracy for both HFrEF and HFpEF, underscoring its value as a scalable tool for early risk stratification^[^43^]^. Collectively, these applications underscore the pivotal role of AI in addressing the most pressing unmet needs in HF care.

The centrality analysis of keywords provides further insights into the structural focus of research on AI in HF. Notably, terms such as risk and mortality emerged as central nodes, underscoring the ongoing emphasis on predicting adverse outcomes and improving survival in this high-risk population. However, the presence of other highly central keywords, including management and prediction, highlights that the research landscape extends beyond risk assessment alone. In particular, management reflects the use of AI to optimize therapeutic strategies, personalize treatment plans, and improve care delivery, while prediction emphasizes the development of models capable of forecasting disease progression, hospital readmissions, and treatment response, thereby supporting proactive and data-driven clinical decision-making.

Recent reviews, such as the work by Cheema *et al*, underscored the expanding role of AI in the management of HF, demonstrating its potential to integrate multimodal data for earlier diagnosis, more precise treatment selection, and personalized care pathways. These applications not only enhance disease monitoring and therapeutic optimization but also facilitate the timely identification of patients progressing to advanced stages. Nonetheless, barriers such as data privacy, workflow integration, and algorithmic fairness remain critical challenges that must be addressed to enable broader clinical implementation^[^31^]^.

Balcioglu *et al* reviewed the emerging role of AI and machine learning in the prediction of right HF (RHF) following left ventricular assist device (LVAD) implantation, one of the most critical complications in advanced HF management. Their analysis showed that AI-based approaches can capture predictive clinical parameters more effectively than conventional risk scores, though current models still demonstrate suboptimal performance due to limited datasets and a lack of prospective validation. Despite these limitations, the review underscored the promise of AI-driven tools to refine perioperative risk stratification, improve patient selection, and ultimately enhance outcomes in this high-risk subgroup^[^44^]^.

We further conducted a cluster analysis and identified nine major research clusters, underscoring the multidisciplinary nature of AI research in HF. Clusters related to cardiac and cardiovascular systems, radiology, and medical informatics highlight the central role of clinical and imaging data in the development of AI-driven diagnostic and prognostic tools^[^19,45,46^]^. The presence of clusters in cell biology^[^47,48^]^, endocrinology^[^49,50^]^, and neurosciences^[^51,52^]^ further illustrates the growing recognition of HF as a systemic and multimodal condition, linking molecular, metabolic, and neural pathways to cardiovascular outcomes. Taken together, these clusters emphasize that the future of AI in HF will rely heavily on cross-disciplinary collaboration, integrating clinical expertise with computational advances to drive precision diagnostics, predictive modeling, and personalized management strategies.

Despite the rapid growth of AI applications for prediction and diagnosis in HF, several barriers continue to limit their translation into routine clinical practice. A key challenge is the gap between algorithmic performance in retrospective or controlled settings and real-world clinical rigor, where data heterogeneity, missing information, and shifting practice patterns can degrade model reliability^[^53–57^]^. In addition, many AI models function as “black boxes,” limiting interpretability and clinician trust, which is essential for adoption in high-stakes decision-making environments. Ethical and legal concerns further complicate implementation, particularly when models are trained on nonrepresentative populations^[^58–60^]^. Moreover, poor integration with existing clinical workflows and electronic health record systems remains a practical obstacle, increasing cognitive burden rather than reducing it^[^61–63^]^.

## Limitations and future directions

This bibliometric analysis has some limitations that should be acknowledged. First, this study relied exclusively on the Web of Science Core Collection, which may have resulted in the omission of some relevant publications indexed in other databases, such as Scopus or PubMed. Prior comparative analyses of citation databases have shown that Web of Science generally captures approximately 80–90% of the citations indexed in Scopus and Microsoft Academic, while Google Scholar provides the broadest coverage but with less stringent source selection criteria. Based on these findings, it is reasonable to estimate that an additional 10–20% of potentially relevant studies on artificial intelligence in HF may exist outside the Web of Science Core Collection. The exclusion of these studies may have led to a modest underestimation of overall publication volume^[^64^]^. However, the core trends, dominant research themes, and leading contributors identified in this analysis are unlikely to be substantially altered, as Web of Science predominantly indexes high-impact, peer-reviewed journals that shape the intellectual structure of the field. Second, although bibliometric methods provide valuable insights into publication trends, citation impact, and collaborative structures, they cannot fully capture the quality, clinical applicability, or translational value of individual studies. Third, citation counts may be influenced by factors such as journal visibility, self-citation, or recency bias, which can distort assessments of true scientific influence. Additionally, keyword-based analyses are limited by author choices and may not fully reflect the evolving conceptual landscape.

Future research should aim to integrate multiple bibliographic databases to provide a more comprehensive view of the field. The application of natural language processing and AI-based text-mining techniques could enhance the depth of bibliometric analyses by enabling semantic clustering of research themes beyond author-provided keywords. Furthermore, longitudinal studies assessing the clinical translation and real-world impact of AI-driven solutions in HF are needed to bridge the gap between academic innovation and patient outcomes. Global collaborations, particularly involving underrepresented regions, will be critical to ensuring diversity of datasets and equity in algorithm development. Finally, the establishment of standardized reporting guidelines, open-access data-sharing initiatives, and robust ethical frameworks will be essential to overcome current barriers and advance the safe, effective, and fair integration of AI into HF care.

## Conclusion

In summary, this bibliometric analysis highlights the rapid growth and evolving landscape of research on AI in HF. Publication trends demonstrate a sharp rise in scientific output over the past decade, reflecting increasing recognition of AI’s potential to transform diagnosis, risk stratification, prognostic modeling, and disease management. The analysis further underscores the pivotal role of leading countries, institutions, and collaborative networks in driving innovation, while also revealing disparities that call for broader global participation. Keyword and cluster analyses confirm that mortality, diagnosis, risk, and prediction remain central themes, with emerging multidisciplinary approaches linking clinical, computational, and biological domains. Collectively, these findings illustrate both the remarkable progress achieved and the ongoing challenges that must be addressed to ensure the effective and equitable translation of AI-driven solutions into clinical practice for heart failure.

## Data Availability

Data can be provided on a reasonable request from the corresponding author.
